# Weighing as part of your care: a feasibility study exploring the re-introduction of weight measurements during pregnancy as part of routine antenatal care

**DOI:** 10.1186/s12884-020-03011-w

**Published:** 2020-05-29

**Authors:** V. Allen-Walker, A. J. Hunter, V. A. Holmes, M. C. McKinley

**Affiliations:** 1 Centre for Public Health, School of Medicine, Dentistry & Biomedical Sciences, Queen’s University Belfast, Institute Clinical Science A, Grosvenor Road, Royal Victoria Hospital, Belfast, BT12 6BA UK; 2 Royal Jubilee Maternity Services, Belfast Health & Social Care Trust, Belfast, UK; 3 UKCRC Centre of Excellence for Public Health (Northern Ireland), School of Medicine, Dentistry & Biomedical Science, Queen’s University Belfast, Belfast, UK

**Keywords:** Pregnancy, Weight measurement, Routine weighing, Antenatal care, Feasibility, Acceptability

## Abstract

**Background:**

The UK does not currently have guidelines on gestational weight gain owing to gaps in the evidence base. Reintroducing routine weighing of women throughout pregnancy would begin to provide the evidence needed to fill this gap. The aim of this research was to re-introduce measurement of weight at each routine antenatal appointment in a small scale study, in order to determine the feasibility and acceptability of implementing the practice on a larger scale.

**Methods:**

A feasibility study, incorporating quantitative and qualitative components, was conducted in one antenatal hospital clinic and with one community midwifery team. Thirty-eight pregnant women were recruited at their 20 week anomaly scan appointment and weighed at their appointments throughout the rest of their pregnancy; five participated in a telephone interview at approximately 37 weeks gestation. Data were collected on: numbers consenting to be weighed, reasons for declining to be weighed and number of weight measurements recorded. Qualitative interviews were used to explore acceptability of the practice to pregnant women.

**Results:**

Overall, 79.2% (38 out of 48) of those approached consented to being weighed throughout pregnancy; of the 10 who declined, three cited not wanting to be weighed. In the interviews, women discussed routine weighing as a positive experience, described several benefits of weighing and indicated they would like more information about weight during pregnancy. No major barriers to the integration of a weight measurement into routine antenatal appointments were encountered. Completion of the weight record sheets that were inserted into women’s handheld notes varied between staff: of the 26 sheets recovered from handheld notes, only 3 (11.5%) had no weights recorded, 17 (65.4%) had between one and three weights recorded and six (23.1%) had more than 4 weights recorded.

**Conclusions:**

In this feasibility study, routine weighing was acceptable to pregnant women. No barriers that would inhibit re-introduction of weighing women throughout pregnancy into standard antenatal care were encountered. Implementation of routine weighing during pregnancy on a larger scale should be considered as it may have benefits for women in the short and long-term, particularly with regard to informing appropriate gestational weight gain guidelines in the UK.

## Background

The consequences associated with overweight and obesity in pregnancy, and excess gestational weight gain (GWG), have been well established, and include increased risks of gestational diabetes, postpartum haemorrhage, caesarean section, increased infant adiposity and shoulder dystocia [1, 2]; Scott-Pillai, Spence, Cardwell, Hunter, & Holmes, 2013). Excess GWG is linked to postpartum weight retention [3] and long-term obesity in women [4]. Although appropriate GWG is central to influencing outcomes and weight trajectories across the childbearing period, there are still no UK specific guidelines on GWG. The National Institute for Clinical Excellence (NICE) did not adopt the Institute of Medicine (IOM) GWG ranges as they are primarily based on observational data from US populations. NICE has highlighted that evidence-based UK guidelines on GWG remain an urgent need (National Institute for Clinical Excellence, 2010) [5].

Reintroducing routine weighing of women throughout pregnancy would begin to provide the evidence needed to fill this gap, and may provide additional clinical benefits [6]. There is a need for contemporary country-specific GWG data on large cohorts of women from all BMI (body mass index) categories, which record patterns of GWG and associations with maternal and infant outcomes, in order to inform guidance on appropriate GWG. Gathering such data in the UK requires a change to current practice in order to reintroduce repeat weight measurements throughout antenatal care as women are currently only weighed at their antenatal booking visit, which takes place between approximately 10–14 weeks gestation. Routine weighing during pregnancy in the UK was phased out during the 1990s, before obesity prevalence rose sharply in the population, in response to the debate that it was no longer clinically useful and that the practice caused anxiety in pregnant women [6, 7]. Previous criticisms that weighing causes anxiety to pregnant women are not upheld by recent qualitative research; evaluations of trials that involve weighing pregnant women have found that women welcome routine weighing and articulate several benefits to the practice [8–10]. Additionally, although qualitative and survey research has found that healthcare professionals are embarrassed to raise the issue of obesity and concerned about causing offence [11–13], a recent feasibility study of routine weighing and setting weight gain targets by community midwives [10] found both women and midwives had no issue with weighing, midwives found no difficulties with broaching GWG, and midwives could integrate the practice into appointments.

Therefore, there is evidence to suggest that integrating routine weighing throughout pregnancy into standard practice in order to provide evidence for UK guidance on GWG is plausible. However, there are practicalities which need to be considered before devoting significant resources to any change in practice, and to explore whether it is feasible to reintroduce routine weighing into standard antenatal care rather than implementing it as part of research study protocols.

The aim of this study was to test the feasibility and acceptability of reintroducing the practice of routine weighing of pregnant women to antenatal care in a small scale study.

The objectives were to:

- Examine the acceptability of routine weighing during pregnancy to women and healthcare professionals.
- Examine to what extent routine weighing can be successfully carried out with intended participants as part of routine antenatal care.
- Examine to what extent routine weighing can be carried out using existing resources.

## Methods

### Study design

Women aged over 18 years old presenting for antenatal care at one hospital clinic (Royal Victoria Hospital Maternity Unity, Belfast) and to one community midwifery team (the Mater Hospital, Belfast, community midwifery team) (as there may be different factors affecting feasibility of routine weighing in the hospital versus community settings) were informed about the study, called ‘Weighing as part of your care’. Women received an information leaflet about the study and, after speaking to the researcher, if they agreed to participate, they provided written informed consent to be weighed and have their weight recorded in their hand-held maternity notes at each antenatal visit throughout their pregnancy. Recruitment for the feasibility study ran for 14 weeks in the hospital setting, and for 7 weeks in the community setting, between April and August 2016. Table one summarises the three main areas of focus of the feasibility study with outcomes of interest and data collection methods, adapted from Bowen et al. [14]. Ethical approval was gained from the National Research Ethics Service NHS Health Research Authority London - City & East Research Ethics Committee Research Ethics Committee REC reference: 15/LO/2070, and research governance approval was provided by the Belfast Health and Social Care Trust and Queen’s University Belfast.

### Design of materials

Materials to support the implementation of routine weighing for the purpose of this feasibility study were designed by the research team and reviewed by senior staff at the Royal Victoria Hospital Belfast. These included: a weight measurement record sheet that was inserted into hand held notes; a study sticker that was placed on the front of the hand-held notes indicating an individual was participating in the study and thus reminding antenatal staff to measure and record weight; and an information sheet on GWG for women that was given to any woman who asked for further information (this was designed by VAW and MM and then reviewed by the clinic’s consultant obstetrician (AH)). The information sheet on GWG contained information on why it is important to eat a healthy, balanced diet during pregnancy, the eating for two myth, the different components of pregnancy weight gain (baby, placenta, amniotic fluid etc) and links to a few useful websites for more information. This information was based on current guidance from the UK National Health Service (NHS) found on the NHS website (www.nhs.uk). Advice on the layout of the weight measurement sheet and which section of the hand-held maternity notes to insert it into was also given by senior midwives from the hospital setting and the consultant obstetrician in Belfast Health & Social Care Trust, as were suggestions on the name of the study.

### Sample and recruitment

All women who attended one antenatal clinic at the Royal Victoria Hospital and those under the care of one community midwifery team from the Mater Hospital were asked to participate in the study, in order to explore the feasibility of the practice in these two different settings that provide antenatal care provision. Given the aims of the study, a sample size calculation was not appropriate. Recruitment was undertaken at the anomaly scan clinic at each of the two settings and all eligible women (i.e. attended the selected antenatal clinic or one of the surgeries providing community midwifery care) were approached to participate in the study. Information sheets were provided to women prior to their scan, and the researcher answered any questions and ascertained interest following the woman’s scan. Written informed consent was taken from those interested in taking part, and a ‘Weighing as part of your care’ sticker and weight measurement record sheet was inserted into the participant’s hand-held maternity notes. Written informed consent covered: consent to be weighed at each appointment; consent to access routinely collected data on pregnancy and birth outcomes from the birth record system; and an option to consent to be contacted at a later date to receive information on participating in an interview about their experiences of being weighed during pregnancy.

Through liaison with senior staff at the Royal Victoria Hospital Belfast and the managers of the Belfast Health and Social Care Trust community midwifery team, one antenatal clinic at the Royal Victoria Hospital Belfast and a number of GP surgeries that host the community midwifery care team were selected to run the feasibility study. Staff providing antenatal care at these locations were introduced to the researcher, and information about the study was provided either at staff meetings or in the workplace. Calibrated portable scales were provided to staff to enable weighing (as women are not routinely weighed and so staff reported a lack of access to weighing scales in the hospital maternity unit and community setting; eight sets were provided). A Standard Operating Procedure was provided to staff, covering how to take and record weight and how to answer any questions they were likely to be asked. Contact details for the researcher were also made available to staff.

### Data collection and analysis

A summary of the data collection methods relevant for the feasibility study outcomes is shown in Table 1.

**Table 1:** Feasibility study outcomes and data collection methods

Area of focus	The feasibility study asks…	Outcomes of interest	Data collection method
Acceptability	To what extent is being weighed at each appointment acceptable to pregnant women? To what extent is weighing pregnant women at each appointment acceptable to maternity staff?	• Satisfaction • Intent to continue use • Perceived appropriateness	• Qualitative interviews with women and health professionals • Question on reasons for declining to be weighed • Numbers consenting to be weighed and access to the maternity care record system
Implementation	To what extent can routine weighing be successfully carried out with intended participants as part of routine antenatal care?	• Degree of execution • Success or failure of execution • Amount, type of resources needed to implement	• Qualitative interviews • Number of completed record sheets • Number of information leaflets provided
Practicality	To what extent can routine weighing be carried out with intended participants using existing means, resources and circumstances, and without outside intervention?	• Factors affecting implementation ease or difficulty • Positive/ negative effects on target participants	• Qualitative interviews • Study records

#### Quantitative data

Records were kept on the numbers of women attending each scan clinic each week, numbers checked for eligibility, and, numbers approached with information about the study, to give an indication of the number who consented to be weighed. Women who declined to participate were asked to complete a short one response question to indicate why they would prefer not to participate in order to provide information on acceptability of the practice. Records were also kept on the number of women who provided consent to access the maternity care record system to indicate the feasibility and acceptability of linking weight measurements to pregnancy outcomes.

An information leaflet on GWG was given to midwives to provide to women who asked whether their weight gain was normal or had other related questions. A known number of leaflets were provided to the antenatal clinic and community midwifery team to allow monitoring of the number of leaflets given to women thus indicating the frequency of questions about GWG as a result of weighing.

Weight recording sheets were retrieved from participants’ hand-held notes after delivery and data regarding number of weights recorded was extracted.

#### Qualitative data collection

Women providing consent to be weighed were asked if they were happy for a researcher to contact them at a later date regarding participating in a telephone interview. The researcher contacted a sample of those who indicated this was acceptable when they were approximately 37 weeks pregnant, with information about the interview and an invite to participate. The interview explored the acceptability of routine weighing to women, and topics included how they felt about being asked to be weighed and the experience of being weighed at each appointment, whether they expected to be weighed, any issues or benefits to being weighed and whether it affected their usual diet and activity levels.

Health professionals involved in the feasibility study were provided with written information about a follow up interview, to explore their experiences of weighing women and their opinions on introducing routine weighing to standard care, and a slip to return their contact details to the researcher if they were interested in participating. This was provided in hard copy format to staff in the staff room of the antenatal clinic, and followed up by visits by the researcher to ascertain interest. Community midwifery staff were emailed an electronic copy of the information sheet, with a follow up email from the researcher to ascertain interest.

Qualitative interviews were transcribed verbatim and fully anonymised; transcripts were analysed using Thematic Analysis [15]. A deductive approach to analysis was used given the aim of considering the acceptability of weighing to pregnant women and health professionals, and thus the transcripts were scrutinised for instances relating to their experience of the study and their opinions on weighing more generally.

## Results

Data presented below are separated by setting: hospital and community.

### Recruitment

Table 2 summarises the recruitment in each setting.

**Table 2:** Summary of recruitment in the hospital and community setting

	Hospital	Community	Total
Provided with information sheet	52	8	60
Provided with information sheet and spoke to researcher	41	7	48
Agreed to participate	32	6	38
Declined:	9	1	10
- In a rush	2		
- Already being weighed/seeing midwife about weight	2	1	
- Did not want to be weighed	3		
- No reason	2		

#### Hospital

Over the recruitment period, 177 women attended the anomaly scan clinic. Of those checked for eligibility (i.e. attended the selected obstetric clinic for antenatal care), 52 of the eligible women were approached with information about the study. Of the remaining 125 women attending for scans: 32 (25.6%) were not eligible owing to pregnancy viability issues; 3 (2.4%) were eligible but were missed before study information could be provided; 2 (1.6%) were eligible but not approached (one was having triplets, and one required an interpreter); and the remaining 88 (70.4%) could not be checked for eligibility before their scan, either due to having no notes, going straight through to their scan before their notes could be checked, or because the researcher was consenting another woman. Of the 52 women who were approached with study information, 51 (98.1%) women accepted information; one (1.9%) declined information as she had several appointments to attend at the hospital that day. Of those who received information about the study (*n* = 51), 10 (19.6%) left before speaking to the researcher or while the researcher was taking consent from another woman. Of the 41 women who spoke to the researcher, 9 declined to participate (22%) and 32 (78%) consented to taking part. Nine (21.9%) declined to take part for the following reasons: too time consuming to give consent as needed to go straight back to work or pick a child up (*n* = 2, 22.2%); already being weighed during pregnancy as part of specialised care they were receiving (*n* = 2, 22.2%); did not want to know weight and/ or did not want to be weighed (*n* = 3, 33.3%); or no reason was given (*n* = 2, 22.2%).

#### Community midwifery team

In total, 41 women attended the selected anomaly scan clinic over the recruitment period. Of these, 10 (24.4%) women were eligible (i.e. they attended one of the selected surgeries for antenatal care) and 8 (80%) were approached with information about the study. All women approached took information about the study. The researcher spoke to seven women; one (12.5%) woman was not approached following her scan to check interest in the study based on advice from the midwife that it would not have been appropriate to do so. Of the seven women who spoke to the researcher, 6 (85.7%) consented to take part. One (14.3%) woman declined to take part in the study because she was waiting to see the midwife after her scan regarding her diet and weight.

All 38 women who consented to take part in the study provided consent for the researchers to access their maternity care record system data and 32 out of 38 (84.2%) women also provided consent to receive information about a follow up interview.

### Provision of information on GWG

At the beginning of the study, the antenatal clinic staff at the hospital were provided with 20 information leaflets on GWG and were informed that these could be given to women if they had any questions about GWG after the weight measurement was taken. At the end, five had been given out (15.6% of the 32 women participating at the hospital site). Community staff were not able to provide information on the number of information leaflets on GWG that were given out.

### Completion of weight measurement record sheets

Of the 38 women who participated in the study, a total of 26 (68.4%) weight record sheets were collected from their maternity hand-held notes. The remaining 12 (31.6%) were missing from notes and unaccounted for. The retrieval rate was 5 out of 6 (83.3%) women participating in the community setting and 21 out of 32 (65.6%) for the hospital setting.

In total, between 0 and 8 weights (mean 2.6 weights), were recorded. To give context to the number of opportunities participants would have had to be weighed: nulliparous women are scheduled to have seven antenatal appointments from the anomaly scan (time of recruitment) to 40 weeks, whereas multiparous women are scheduled to have four antenatal appointments. In the hospital setting, a total of 21 record sheets were available for participating women. Of these, considering both the second and third trimesters, three (14.3%) had no weights recorded at all, 14 (66.6% had between one and three recorded weights, and four (19.1%) had four or more recorded weights. In the community setting, there were five record sheets available for participating women. Of these, across both the second and third trimesters, three women (60%) had between one and three weights recorded, and two (40%) had four or more recorded weights. Overall, for the 26 sheets retrieved from notes: three (11.5%) had no weight recorded, 17 (65.4%) had between one and three weights recorded and six (23.1%) had more than four weights recorded.

### Interview recruitment

Of the 32 women who consented to receiving information about a follow up interview, a total of 28 were contacted: one left no contact details and three were missed during their follow up period due to the researcher being unavailable during these weeks. Of the remaining 28 women who were contacted, three left incorrect contact information, one declined to take part, one expressed interest but did not respond to suggested interview slots, one arranged an interview slot but did not answer the phone and 17 did not respond. This left a sample of five women who took part in a telephone interview.

Information about the interview was provided to healthcare staff, however, no staff contacted the researcher, despite reminders, about taking part and therefore there are no interviews with healthcare professionals involved in the feasibility study.

### Demographics of women who took part in interviews

A total of five women, mean 34 years (standard deviation 3.16 years) participated in a follow up telephone interview when they were between 36 and 38 weeks pregnant, and all had been weighed in the hospital setting. Four (80%) were married, four (80%) had one other child, three (60%) had been educated to degree level or above, one (20%) to Diploma level and one (20%) to GCSE (General Certificate of Secondary Education) and all were employed full-time. Two women indicated they had been weighed at every antenatal appointment and three indicated they had been weighed at most appointments.

### Qualitative findings

Deductive thematic analysis of the interview transcripts resulted in the development of three themes relating to the acceptability and feasibility of routine weighing: *Routine weighing was a positive experience; Routine weighing could be part of antenatal care; and Women would like some information about weight during pregnancy.* These themes are discussed below, with illustrative quotes recorded in Table 3.

**Table 3:** Illustrative quotes for themes

**Routine weighing was a positive experience**	*During pregnancy, I was just, I just think it was, it’s, it’s good to monitor it (weight) so… they can check if there’s any underlying health issues during your pregnancy as you’re excessively gaining weight or not….. I think it is helpful and I didn’t have a problem with being weighed during pregnancy at all.*
	*I’ve found it quite good just like each appointment I’m getting weighed each time so I’m like it’s in my mind set I’m just thinking about it a wee bit more whereas before, the first one I wasn’t thinking about it at all I was just like, oh I’m pregnant it’ll be the only time I have an excuse not to be worrying about my weight to be honest.*
	*It was a case of going to my ante-natal appointments, getting my blood pressure checked, getting my urine checked and getting my weight done so it was just another part of you know the monitoring really I suppose.*
	*Yes I was interested – I was interested to see basically because I would be expecting it to increase as the baby grew, which, which it has you know. It is pretty much I feel as if it’s all baby weight really on me. So yeah it was always interesting to see how much that has gone up by.*
	*I suppose it just sort of made you realise that you were being weighed and that um I suppose you do try to be healthy, as healthy as you can, and eat healthily and not eat as much sort of junk food or whatever.*
	*I have been watching very closely and thinking my goodness right listen I need to do a little bit more walking here or I need to not eat so many lollies cause I’m craving ice lollies you know so….*
**Routine weighing could be part of antenatal care**	*I mean it was good to kind of know what you were putting on so it would be something that I think should be involved like every pregnancy like…at every appointment…‘cos some people kind of think aye you’re eating for two.*
	*I think… yeah, I do think they should at least mention it (weight), particularly if it’s going to change like you know ……if you actually go and read about it, and it says all about healthy weight gain, and I think that the book they give you, the NHS books they give you at booking in, you know it says about how important it is but it’s not mentioned so you know it’s really, if it is that important, then they should be mentioning it a lot more…*
	*Yeah unless you’re told at your booking scan that you’re going to be weighed at the start and end of your pregnancy but that information isn’t provided at your booking scan you’re just asked for your height and then you’re weighed, but I don’t see the point of them weighing you at the start of your pregnancy because if it’s nothing to report on at the end, I don’t see why you would weigh someone at the start…you need a series of data over time.*
	*It is something that definitely you can’t just ignore because by the end of your pregnancy you know you could end up putting on a serious amount of weight which could lead to health problems with the baby, in pregnancy and afterwards you know so it is something that needs to be you know I feel monitored.*
	*I think they should, if they’ve (health professionals) no objection, I don’t see why not. I think anything that can be measured throughout your pregnancy and monitored is excellent you know, every time you go you have an appointment, a medical appointment with your midwife or your consultant or whatever, you always get your blood pressure and urine.*
	*Its always done at the first appointment so I don’t see why you wouldn’t have a few milestones throughout your pregnancy that you should be weighed as well…you know you’ve various points over time and the pregnancy where weight can be monitored I think it would be helpful.*
	*I think it’s a good idea (measuring weight in pregnancy) and think that if it can be continued it should be continued, but again I’m mindful of the pressures I suppose of healthcare staff and it’s probably another thing on their lists to add to, and I think the appointments are very… you know there’s a lot to be done in the appointments you know and there’s a lot to be done and to have a queue of people outside to come and be weighed…but it literally I mean weighing someone takes 2 s…*
	*It hasn’t been an issue (being weighed), I think to be honest…It’s sort of like they’re (health professionals) just sort of ticking all the boxes and trying to get done what they have to get done and then, you know. it’s not really, no it (weighing women) wouldn’t be a huge priority of theirs I don’t think.*
**Women would like some information about weight during pregnancy**	*No Google gave me that (information on weight gain in pregnancy)…Just sort of checking out you know online of what was you know for each wee check-ups ….what was too much, what wasn’t enough, you know just to make sure that everything was fine and it was a healthy, healthy weight that you were putting on and not too much too soon you know. Plus obviously pregnancy books and things like that do you know you would sort of check those to make sure that you weren’t you know, putting on too much.*
	*Um I suppose I…I suppose a wee bit of information would’ve been yeah would’ve been nice to have a wee bit of information I suppose, I’m from a health professional background myself so I’ve a wee bit of knowledge anyway you know so I suppose that helps but maybe somebody who wouldn’t have that knowledge it might be, might be helpful for them*
	*I suppose like I was under thirties (BMI at booking) but you know you they sort of said you know you’re at risk of maybe gestational diabetes and I was thinking well maybe mine (BMI) normally about 24…Aha and you know I’m thinking okay… it was quite confusing to work out whether I was at risk because I put so much weight on in the first trimester or whether they felt that that was my pre-pregnancy weight and that’s what I had been carrying normally?*
	*Yeah unless you’re told at your booking scan that you’re going to be weighed at the start and end of your pregnancy but that information isn’t provided at your booking scan you’re just asked for your height and then you’re weighed, but I don’t see the point of them weighing you at the start of your pregnancy because if it’s nothing to report on at the end, I don’t see why you would weigh someone at the start…you need a series of data over time.*

#### Routine weighing was a positive experience

Participants described being weighed during their antenatal appointments as ‘just another part of monitoring’ and indicated it was a positive experience in many ways. For the majority who were satisfied with their weight gain, routine weighing served as a point of interest or reassurance. It was felt that a certain amount of weight gain was both necessary and important for a healthy pregnancy, and that regular weighing provided reassurance that the baby was growing; indeed the weight measurements were of interest in relation to the development of the pregnancy. One participant felt that awareness of her weight enabled her to make healthy behaviour changes such as increasing walking and decreasing unhealthy snacking and another associated being weighed with heightened awareness of the need to eat healthily.

All participants reported that they had no issue with being weighed during their pregnancies; one commented that although women in general might feel a bit anxious about being weighed it was still good to be aware of pregnancy weight gain. Another reflected that weight gain generally has negative connotations in society and that women tend to be concerned about their weight, but again felt regular weighing during pregnancy was of benefit. Some monitored their weight both before and during pregnancy, whereas others did not feel they needed to do so; thus being weighed was acceptable to women who were generally more aware about their weight and for those where it was not an issue. Additionally, none of the participants reported any negative effects as a result of routine weighing and considered the practice to fit in well as part of their routine antenatal checks as discussed further in the next theme.

#### Routine weighing could be part of antenatal care

All women interviewed felt there was no reason why routine weighing should not be part of antenatal care and discussed how they felt the practice could be beneficial to all women, expressing confusion as to why it was not already implemented. Weighing was considered to be beneficial in monitoring potential health consequences and raising the issue of excess GWG, enabling women to ‘keep an eye’ on their own weight gain. Some discussed previous experiences of struggling to lose weight postpartum, sometimes after gaining a lot of weight during pregnancy, and reflected that an awareness of GWG was key to minimising this struggle. Additionally, the concept of ‘eating for two’ during pregnancy was discussed, which was perceived to be used by pregnant women as a reason to relax healthy lifestyle habits and suggests that this myth is still prevalent; weighing was seen as an avenue to address this and other relevant lifestyle behaviours. Participants considered that GWG was not a current priority for healthcare professionals and felt this might be why it was not currently addressed; women were mindful of the pressures facing antenatal care staff but felt that it would not take long to integrate a weight measurement into current health checks.

#### Women would like some information about weight during pregnancy

None of the participants reported receiving information on GWG as a result of being weighed, and they had no issue with this. This was generally because they felt they did not have a weight issue, or had a good background understanding already. Indeed, some sought out information on GWG themselves as a result of study participation and out of existing interest in the topic. However, it was acknowledged that some information on GWG could be useful for those who did not have any background knowledge. Women expressed a desire for information on why weight was currently measured at booking but not at later antenatal appointments: one participant could not understand the point in taking a weight at the booking appointment at the beginning of pregnancy; another participant expressed frustration that her booking weight (measured at around 12 weeks gestation) was recorded as her pre-pregnancy weight but she had gained a significant amount of weight before the booking appointment and so felt her booking BMI (overweight range) did not reflect her usual BMI (normal range). Women also expressed that they did not understand why the issue of GWG was not broached by their healthcare providers as it was mentioned in the literature they were given at booking.

## Discussion

This study aimed to explore the feasibility and acceptability of reintroducing routine weighing of pregnant women at each antenatal appointment in both the hospital and community setting. Few women declined to be weighed suggesting that routine weighing during pregnancy is acceptable to the vast majority of women and this is supported by the qualitative findings which indicated that women found being weighed during pregnancy a positive experience and felt it could be made part of standardised care. Routine weighing was phased out in the UK during the 1990s before obesity prevalence rose sharply in the population [6, 7]. There is now arguably a clinical and epidemiological imperative to reconsider the practice given the current context of high obesity rates and the issues surrounding this have been discussed previously [6]. The findings of this study further inform the debate on routine weighing during pregnancy by providing evidence that it does not cause anxiety in pregnant women and, indeed, women appeared to welcome it and discussed several benefits of the practice. These included reassurance that the baby was growing, heightened awareness of lifestyle behaviours such as being more active and paying attention to snacking, and heightened awareness of having to lose weight gained after the baby is born. This supports previous findings that postnatal women who were weighed throughout pregnancy as part of a study felt it should be part of standard care, perceived it to have several benefits, and did not experience anxiety or negative effects as a result of being weighed [8]. These findings also give tentative support to those of Daley et al. [10] who found weighing was acceptable to pregnant women in a community setting; however, Daley et al. [10] were also conducting an intervention that included setting GWG goals and providing individualised advice on weight management in addition to conducting regular weight measurements in pregnancy. Additionally, the findings presented here support those of Brownfoot and colleagues [9], who found that women participating in a randomised controlled trial in Australia welcomed the reintroduction of routine weighing, and did not feel anxious as a result of being weighed. All the participants in the present study also gave consent for the researchers to access their maternal health data, suggesting it is feasible to link GWG data to pregnancy and birth outcomes in future health research. Therefore, the previously cited barrier of potentially causing anxiety in pregnant women lacks empirical evidence, and, on the contrary, women are positive towards the concept of routine antenatal weighing.

For the purpose of this feasibility study, weight record sheets were inserted into hand-held antenatal notes and a sticker was added to the notes to indicate to healthcare staff that a woman was participating in the ‘Weighing as part of your care’ study. The completion of weight record sheets varied in terms of number of weights recorded but, overall, out of the 26 sheets retrieved from notes, only three women had no weights recorded, most women (*n* = 17) had between one and three weights recorded and six women had more than four weights recorded. Twelve sheets were missing from hand held maternity notes. These results suggests some variation in the extent to which healthcare staff were weighing women, possibly reasons for this may be that staff were unable to identify participating women or staff were unfamiliar with the study and what was required in the SOP or staff did not have scales available during appointments. All of these factors relate to the research process rather than the actual process of weighing women. Weighing women as part of a research study presents a different scenario than if this was part of routine antenatal data collection. In the latter case, women would not need a sticker in their notes to flag to staff to weigh them as *all* women would be weighed, and scales would be readily available in all antenatal clinics for this purpose. We attempted to collect views of staff through interviews but, unfortunately, there was no uptake from staff to participate in an interview, due to a lack of reported time to attend. Field notes recorded by the researcher after informally discussing the study with staff suggest that they were happy to weigh women; however, in the hospital setting in particular, the scales we provided were not always set out for the identified clinic every week. Aside from making weighing scales more widely available in antenatal settings, there are no other major costs associated with re-introducing weighing throughout pregnancy.

A pilot observational study provides insights to the feasibility of conducting weight measurements in a large cohort (*n* = 824); the ‘Fit for Birth’ study aimed to describe patterns of GWG in obese women and explore associations of pregnancy weight change with health outcomes [16]. The researchers found that just over 50% of their sample had weights recorded in all three of the study periods, and reflected that staff may not prioritise measurements that aren’t deemed clinically necessary, especially when no incentive is provided [16]. Furthermore, Narayanan and colleagues reflected that introducing weight measurements for all women may have improved completed data sets, as midwives may have been worried about stigmatising women with increased BMIs [16]. Since weighing was only operational in some antenatal clinics for the research presented here, identifying the women who were participating (via the sticker on handheld notes) may have added extra burden and resulted in less weights being recorded for some participants. Weighing as part of routine antenatal care, with space to record weights incorporated as standard fields into current hand-held notes, would be very different to the processes required in a research setting; midwives and healthcare assistants are likely to be more compliant if it is ‘expected’ of them clinically. However, consideration of whether health professionals feel well placed to address the topic of weight gain during pregnancy also requires consideration. Recording a woman’s weight during pregnancy presents an opportunity to have a conversation with women about this potentially sensitive subject and previous studies indicate that midwives may avoid these conversations for many reasons [11, 17]. Women in this study expressed dissatisfaction that no explanation is currently provided to women about the decision to only weigh women once at the booking appointment and how this weight is then used to determine their risk category. Women would like more open communication with health professionals on the topic of weight and pregnancy and, indeed, have indicated they expect the issue to be raised [8, 9]; however, if routine weighing throughout pregnancy was re-introduced, training would be required to ensure existing and future staff felt comfortable addressing the issue.

### Strengths and limitations

A strength of this feasibility study is the focus on reintroducing repeat weight measurements to standard antenatal care as opposed to as part of an intervention package, which presents a unique contribution to the area. Women’s views on the practice were also considered in-depth, and data on processes relevant to implementing the practice on a wider scale collected. However, feedback from women was only received from those in the hospital setting and so there is no data available on the experiences of women in the community setting. Furthermore, it was not possible to obtain formal feedback from health professionals involved in the study due to constraints on staff time to participate in an interview. Alternative methods, such as brief, anonymised questionnaires or group feedback at staff meetings, may prove more fruitful in the future as a way capturing the opinions of those conducting weight measurements. This, and the fact that not all weight measurements sheets were retrieved from notes are potential sources of bias in the study.

Since the aim of the study was to integrate weighing during pregnancy into routine antenatal care appointments, health professionals were not asked to collect ‘research’ data, such as BMI, parity and demographics, as this would have been burdensome for the health professionals and would not have mimicked real-world implementation. Without this data it is not possible to comment on the representativeness of the sample reported here. However, all women attending the antenatal clinics involved were eligible and few women declined.

A higher number of women could potentially have been captured during the timeframe of the study, however, recruitment was constrained by several factors. At the anomaly scan clinic, it was not possible to check all women for study eligibility due to the constraints of recruiting in a busy clinic; sometimes women were running late so went straight through to their appointment, or conversely the clinic was running ahead of time and women were called straight in, so there was no time to approach women with study information before their scan. Furthermore, in this setting, women were required to read a consent form for the anomaly scan before their appointment, meaning there was not always time to provide study information too. In both settings, some women who were provided with information about the study were missed leaving the clinic after their scan, as the researcher was taking consent from other women in a different room. Of the ten who declined, two stated they did not have enough time to complete the consent forms, supporting the notion that the logistics of the research process impacted on recruitment numbers. Ideally, for a research study such as this having another person to help with recruitment would have helped to increase numbers but this was not possible. Many of the limitations encountered here would not apply if routine weighing was part of routine antenatal care rather than being explored in this research context.

### Future directions

The data presented here demonstrate routine weighing to be feasible and acceptable to women. The main issues encountered in this feasibility study were attributable to the research process rather than the task of weighing women at an antenatal appointment. Implementation of weighing women throughout pregnancy on a wider scale would help inform the debate about whether the IOM guidelines on GWG are appropriate for the UK and Europe. Additionally, it would enhance our understanding of the clinical utility of weighing women in relation to informing clinical decisions in our society where overweight and obesity dominate but the situation of underweight in pregnancy and inadequate gestational weight gain also exists. A background of routine weight measurements throughout pregnancy would also assist with implementation and evaluation of lifestyle based interventions for weight management before, during and after pregnancy.

## Conclusion

In this study, routine weighing during pregnancy was acceptable to pregnant women, and feasible to reintroduce into current antenatal care but would require investment in the form of provision of weighing scales at antenatal clinics. Implementation of routine weighing during pregnancy on a larger scale should be considered as it may have benefits for women in the short and long-term particularly with regard to informing appropriate gestational weight gain guidelines in the UK.

## Data Availability

The datasets used and/or analysed during the current study are available from the corresponding author on reasonable request.

## Abbreviations

BMI: Body mass index
GWG: Gestational weight gain
IOM: Institute of Medicine
NICE: National Institute of Clinical Excellence
